# The TFPI2–PPARγ axis induces M2 polarization and inhibits fibroblast activation to promote recovery from post-myocardial infarction in diabetic mice

**DOI:** 10.1186/s12950-023-00357-8

**Published:** 2023-11-01

**Authors:** Mengqi Guo, Zongyi Xia, Yefeng Hong, Hongwei Ji, Fuhai Li, Wenheng Liu, Shaohua Li, Hui Xin, Kai Tan, Zhexun Lian

**Affiliations:** Department of Cardiology, The Affiliated Hospital of Qingdao University, 16 Jiangsu Road, Qingdao, 266003 Shandong China

**Keywords:** Tissue factor pathway inhibitor-2, Diabetes mellitus, Myocardial infarction, Fibroblast migration, MMPs, Collagen, Fibrosis, Macrophage polarization

## Abstract

**Background:**

Diabetes mellitus is one of the causes of poor ventricular remodelling and poor cardiac recovery after myocardial infarction (MI). We previously reported that tissue factor pathway inhibitor-2 (TFPI2) was downregulated in response to hyperglycaemia and that it played a pivotal role in extracellular matrix (ECM) degradation and cell migration. Nonetheless, the function and mechanism of TFPI2 in post-MI remodelling under diabetic conditions remain unclear. Therefore, in the present study, we investigated the role of TFPI2 in post-MI effects in a diabetic mouse model.

**Results:**

TFPI2 expression was markedly decreased in the infarcted myocardium of diabetic MI mice compared with that in non-diabetic mice. TFPI2 knockdown in the MI mouse model promoted fibroblast activation and migration as well as matrix metalloproteinase (MMP) expression, leading to disproportionate fibrosis remodelling and poor cardiac recovery. TFPI2 silencing promoted pro-inflammatory M1 macrophage polarization, which is consistent with the results of TFPI2 downregulation and M1 polarization under diabetic conditions. In contrast, TFPI2 overexpression in diabetic MI mice protected against adverse cardiac remodelling and functional deterioration. TFPI2 overexpression also inhibited MMP2 and MMP9 expression and attenuated fibroblast activation and migration, as well as excessive collagen production, in the infarcted myocardium of diabetic mice. TFPI2 promoted an earlier phenotype transition of pro-inflammatory M1 macrophages to reparative M2 macrophages via activation of peroxisome proliferator-activated receptor gamma.

**Conclusions:**

This study highlights TFPI2 as a promising therapeutic target for early resolution of post-MI inflammation and disproportionate ECM remodelling under diabetic conditions.

**Supplementary Information:**

The online version contains supplementary material available at 10.1186/s12950-023-00357-8.

## Background

Diabetes mellitus (DM) constitutes one of the largest emerging threats to health in the twenty-first century; by 2030, as many as 360 million people world-wide are estimated to be affected [1]. Patients with DM have a three-fold increased risk of acute myocardial infarction (AMI), which usually occurs 15 years earlier than in non-DM patients [2, 3]. Moreover, the mortality rate of patients with DM in the acute phase of MI or during long-term follow-up remains twice as high as that in patients without DM [4, 5]. The underlying mechanisms may be related to prolonged inflammation induced by hyperglycaemia, excessive degradation of the extracellular matrix (ECM), and disproportionate fibrosis remodelling in the infarcted myocardium, leading to poor left ventricular remodelling and reduced cardiac function [6]. Therefore, it is essential to minimize adverse cardiac remodelling in post-MI patients with DM [7].

In the first few hours after the onset of ischemia, neutrophils accumulate in the infarcted myocardium. Thereafter, monocytes and macrophages dominate the cellular infiltrate, wherein the monocytes differentiate into pro-inflammatory M1 macrophages, which are responsible for clearing necrotic cells and debris and are an important source of matrix metalloproteinases (MMPs) for ECM degradation [8]. This is followed by a repair phase dominated by reparative M2 macrophages producing anti-inflammatory factors, as well as the proliferation, differentiation, and migration of cardiac fibroblasts (CFs) [9, 10]. In accordance with this observation, delayed transition of pro-inflammatory M1 to reparative M2 macrophages has been observed in the diabetic environment, leading to a prolonged inflammatory phase [11–13]. The triggered inflammation and neurohumoral dysfunction during MI and diabetic insult also promote endothelial–mesenchymal transition (EndMT), a process by which endothelial cells transition into mesenchymal cells, such as CFs and smooth muscle cells [14]. Moreover, CF activation and migration play a vital role in the synthesis and deposition of matrix proteins, particularly collagens, leading to tissue remodelling [15, 16]. Therefore, appropriate modulation of macrophage polarization and CF function may be crucial for improving post-MI cardiac remodelling in patients with DM.

We previously reported that tissue factor pathway inhibitor-2 (TFPI2) was downregulated in response to hyperglycaemia at least partially via DNA hypermethylation of its promoter [17]. As a broad-spectrum Kunitz-type inhibitor, TFPI2 is considered a critical regulator of MMP expression and activity and ECM degradation [18]. Moreover, TFPI2 may play a role in macrophage polarization and inflammatory responses. In lipopolysaccharide (LPS)-stimulated macrophages, TFPI2 levels gradually decrease, suggesting that TFPI2 downregulation may be associated with the activation of pro-inflammatory M1 macrophages [19]. TFPI2 activates peroxisome proliferator-activated receptor gamma (PPARγ), which plays an important role in inducing M2 macrophage polarization and anti-inflammatory responses [20, 21]. TFPI2 is reportedly involved in EMT in diabetic nephropathy and plays a major role in tissue fibrosis [22]. However, whether TFPI2 plays a role in post-MI remodelling remains unclear. Therefore, the present study aimed to investigate the role of TFPI2 in fibroblast activation and migration, collagen synthesis, MMP expression, M1/M2 polarization, and related signalling in regulating post-MI inflammation and ventricular remodelling in a diabetic mouse model.

## Results

### Hyperglycaemia downregulates TFPI2 expression in the infarcted myocardium resulting in poor functional recovery during post-MI remodelling

**Fig. 1:**
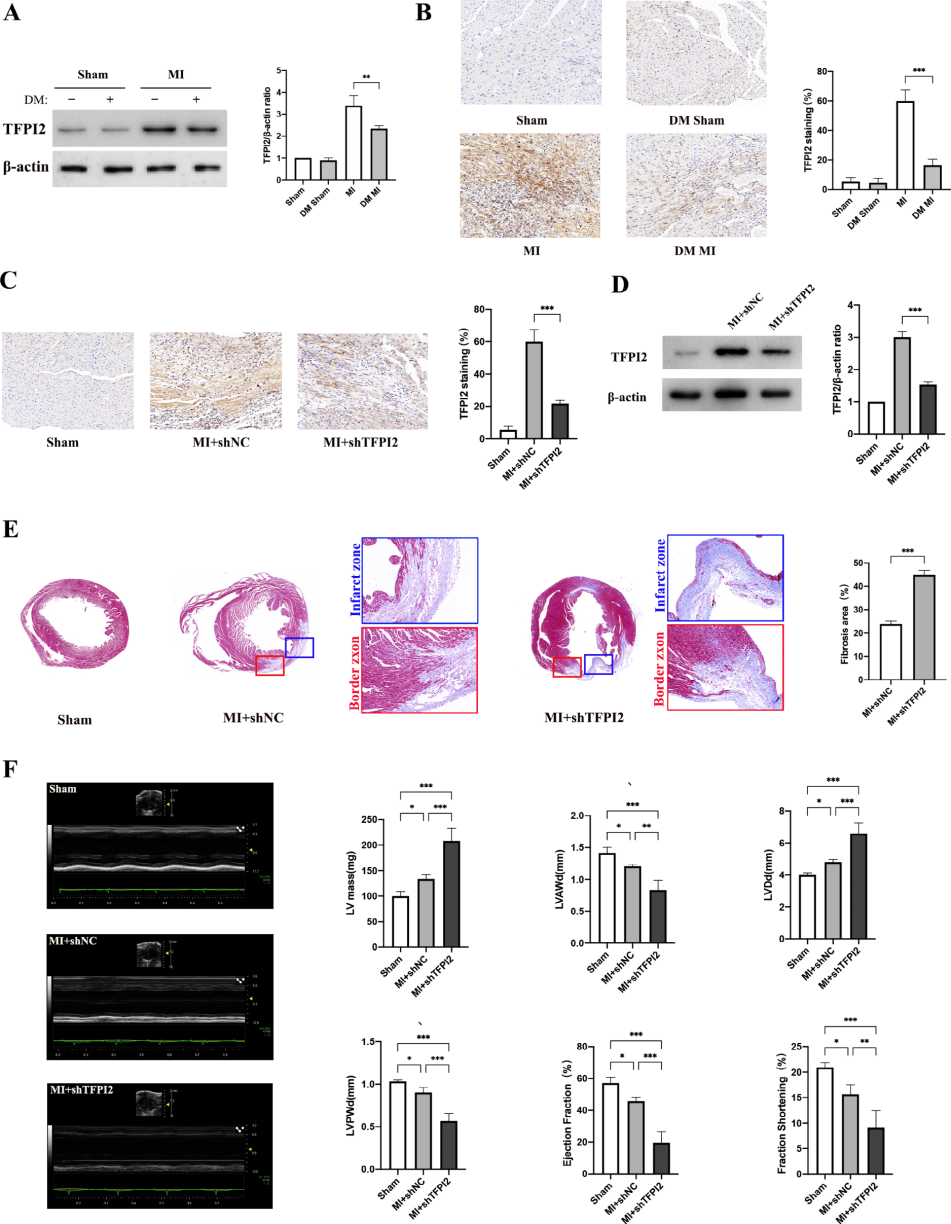
Effects of TFPI2 knockdown on cardiac remodeling and function recovery after MI (**a**, **b**) Western blot analysis and immunohistochemical staining of TFPI2 in the myocardium of mice at 3 weeks after MI (n = 3). (**c**, **d**) Western blot analysis and TFPI2 staining to verify TFPI2 knockdown. (**e**) Images of the infarct and border zones after Masson’s trichome staining of the myocardial samples at 3 weeks after infarction, and quantitative analysis of the fibrosis area (n = 3). (**f**) Quantitative analysis of function recovery using echocardiography (n = 4). Sham, sham operation (magnification: ×100); MI, myocardial infarction; DM, diabetes mellitus; sh-TFPI2, sh-TFPI2 transfection; sh-NC, sh-NC transfection. Data represent mean ± standard deviation (SD). Data were analyzed using one-way ANOVA and Tukey’s post hoc test. ^*^P < 0.05, ^**^P < 0.01, ^***^P < 0.001

The expression of TFPI2 was similar between the sham-ND and sham-DM groups, enhanced in the MI-DM group compared to the sham-DM group, and substantially decreased in the MI-DM group compared to the MI-ND group, 3 weeks post-infarction (Fig. 1a, b).

Consequently, we explored whether TFPI2 downregulation plays a role in post-MI cardiac remodelling. We first established an MI mouse model with TFPI2 knockdown and then verified TFPI2 silencing in the myocardium of mice 3 weeks after infarction (Fig. 1c, d). The area of fibrosis remodelling at both the infarct and border zones was substantially larger in the sh-TFPI2 group than in the sh-CNC group, as revealed by Masson’s trichome staining (Fig. 1e). Moreover, the mice of the sh-TFPI2 group exhibited a higher reduction in left ventricular end-diastolic anterior wall thickness (LVAWd) and left ventricular end-diastolic posterior wall thickness (LVPWd), greater enlargement of left ventricular end-diastolic internal dimension (LVDd) and left ventricular (LV) mass, and a consistently lower ejection fraction (EF) and fraction shortening (FS), than in the sh-NC group, suggesting poor functional recovery of the infarcted heart in the sh-TFPI2 group (Fig. 1f).

### Knockdown of TFPI2 promotes fibroblast activation and M1 macrophage polarization in post-MI remodelling

**Fig. 2:**
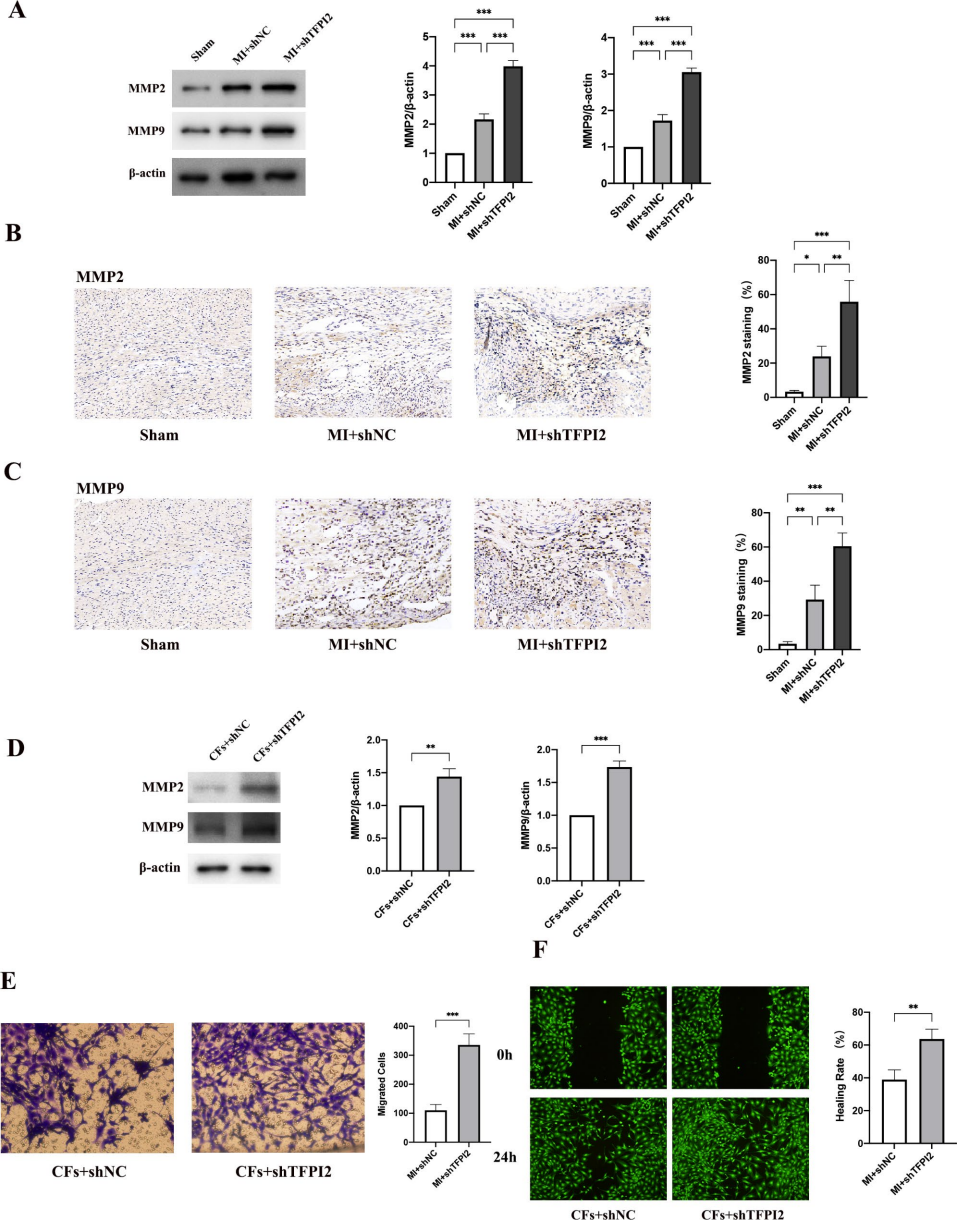
Effects of TFPI2 knockdown on MMP expression, fibroblast activation, and migration after MI (*a*) Western blot analysis of MMP2 and MMP9 in the infarcted heart tissue samples and their quantitative analysis (n = 3, respectively). (**b**, **c**) Representative staining of matrix metalloproteinase 2 (MMP2) and MMP9 in the infarcted heart tissue samples and their quantitative analysis (n = 3). (**d**) Western blot analysis of MMP2 and MMP9 in CFs transfected with sh-TFPI2 (n = 3, respectively). (**e**, **f**) Wound healing and Transwell assays for determination of CF migration. (**e**) CFs on the external surface of the Transwell chambers were stained using crystal violet and imaged under a microscope (magnification: ×200). (**f**) Wounds were made through the cell monolayer and visualized under a microscope after 24 h. MI, myocardial infarction; sh-NC, sh-NC transfection; sh-TFPI2, sh-TFPI2 transfection. Data represent mean ± standard deviation (SD). Data were analyzed using one-way ANOVA and Tukey’s post hoc test. ^*^P < 0.05, ^**^P < 0.01, ^***^P < 0.001

Consistent with a previous study [23], we found that the expression of MMP2 and MMP9 was notably increased in the infarcted myocardium of the sh-TFPI2 group, compared with that in the sh-NC group (Fig. 2a–c). As macrophages and fibroblasts are the main source of MMPs in the infarcted heart [24], we determined whether TFPI2 could inhibit MMP expression in CFs or bone marrow-derived macrophages (BMDMs). The expression of MMP2 and MMP9 was higher in both CFs and BMDMs transfected with sh-TFPI2 than that in the sh-NC groups (Fig. 2d, Supplementary Fig. 1 of Additional File 1). Moreover, Transwell migration and wound healing assays revealed that TFPI2 knockdown substantially promoted CF migration (Fig. 2e, f). The TFPI2 knockdown promoted excessive MMP production and CF activation and migration, which may contribute to disproportionate ECM degradation and fibrosis remodelling in the infarcted heart.

**Fig. 3:**
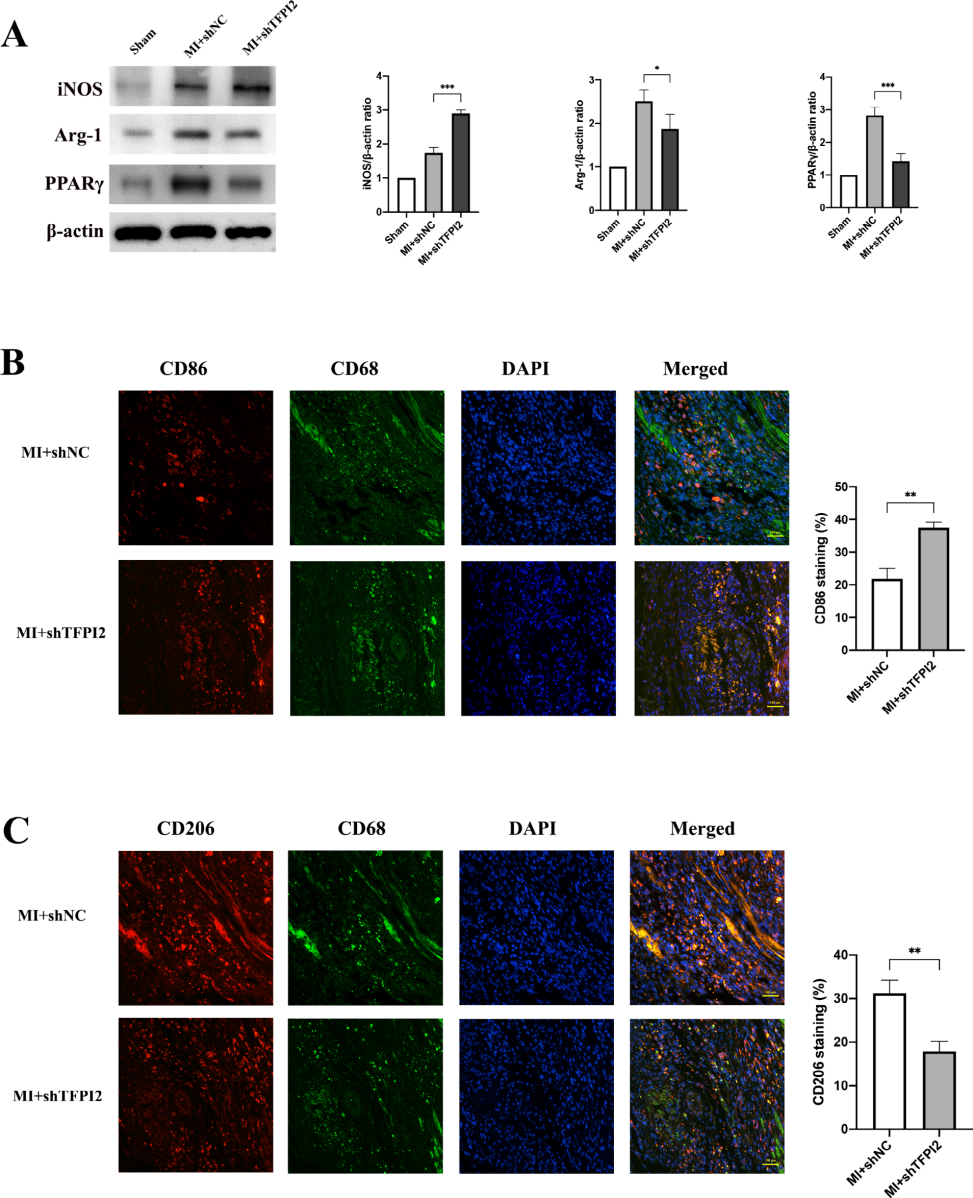
Effects of TFPI2 knockdown on macrophage polarization in post-MI myocardium (**a**) Western blot analysis of iNOS, Arg-1 and PPARγ at 3 weeks after infarction (n = 3). (**b**) Co-immunofluorescence staining of the macrophage marker (CD68, green) and M1 macrophage marker (CD86, red) of infarcted myocardium samples (n = 3). (**c**) Co-immunofluorescence staining of the macrophage marker (CD68, green) and M2 macrophage marker (CD206, red) of infarcted myocardium samples (n = 3). Sham, sham operation; MI, myocardial infarction; sh-TFPI2, sh-TFPI2 transfection; sh-NC, sh-NC transfection. Data represent mean ± standard deviation (SD). Data were analyzed using one-way ANOVA and Tukey’s post hoc test. ^*^P < 0.05, ^**^P < 0.01, ^***^P < 0.001

M1 macrophages are a major source of MMPs for ECM degradation and inflammation [8]. Hence, we investigated whether TFPI2 could regulate macrophage polarization in the infarcted heart. Western blot analysis revealed substantially increased inducible nitric oxide synthase (iNOS) levels and decreased arginase-1(Arg-1) and PPARγ levels in the sh-TFPI2 group compared to the control. We then used double immunofluorescence staining to identify M1 and M2 macrophages in the infarcted myocardium, which revealed that the mice in the sh-TFPI2 group exhibited a higher number of CD86^+^ macrophages than those in the sh-NC group, 3 weeks after infarction (Fig. 3a). Conversely, the number of CD206 + macrophages decreased in the TFPI2 knockdown group compared to that in the sh-NC group (Fig. 3b). TFPI2 knockdown promoted M1 macrophage polarization and MMP production.

### TFPI2 overexpression inhibits activation and migration of fibroblast and collagen deposition in post-MI remodelling in DM

**Fig. 4:**
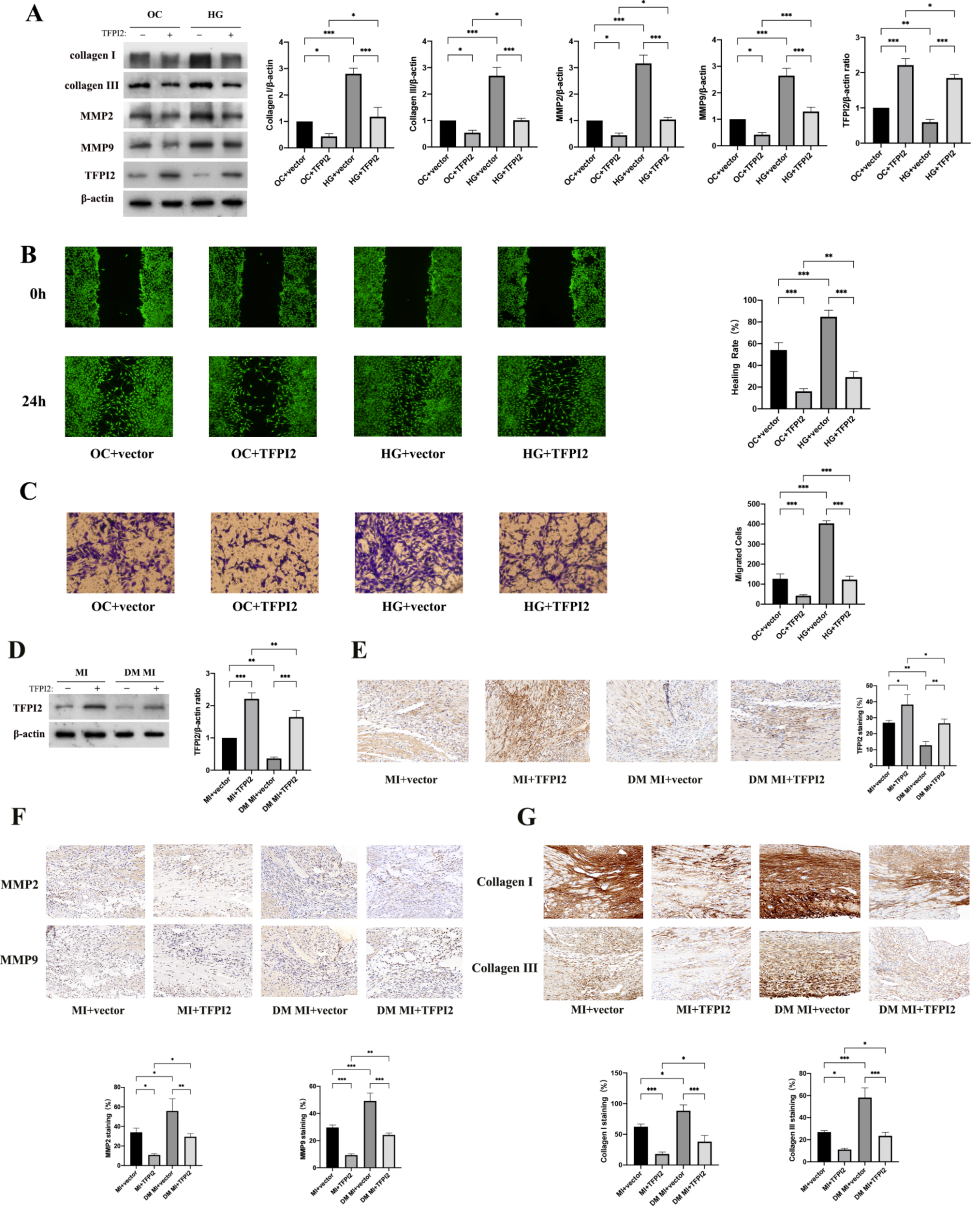
Effects of TFPI2 overexpression on fibroblast activation, migration, and collagen deposition in diabetic MI mice (**a**) Western blot analysis of MMP2, MMP9, collagen I, and collagen III in CFs (n = 3). (**b**, **c**) Wound healing and Transwell assays for determination of cell migration. (**b**) Wounds were made through the cell monolayer and imaged using a microscope at 24 h (magnification: ×100). (**c**) CFs on the external surface of the Transwell chambers were stained using crystal violet and imaged using a microscope (magnification: ×200). (**d**, **e**) Western blot analysis and representative staining of TFPI2 to verify TFPI2 overexpression in myocardium samples (n = 3). (**f**) Representative staining of MMP2 and MMP9 in the infarcted myocardium samples and their quantitative analysis (n = 3). (**g**) Representative staining of collagen I and collagen III in the infarcted myocardium samples and their quantitative analysis (n = 3). MI, myocardial infarction; DM, diabetes mellitus; TFPI2, transfection with TFPI2 cDNA; vector, transfection with the empty vector. Data represent mean ± standard deviation (SD). Data were analyzed using one-way ANOVA and Tukey’s post hoc test. ^*^P < 0.05, ^**^P < 0.01, ^***^P < 0.001

We investigated the role of TFPI2 in high-glucose (HG)-stimulated CF activation, migration, and collagen production. Western blot analysis indicated that the expression of TFPI2 in CFs was substantially downregulated under HG stimulation. HG treatment increased MMP2 and MMP9 expression, which was reversed by TFPI2 overexpression (Fig. 4a). Consistently, wound healing and Transwell migration assays demonstrated that TFPI2 overexpression attenuated HG-induced CF migration (Fig. 4b, c). We observed that TFPI2 overexpression attenuated HG-induced increase in collagen I and III expression (Fig. 4a).

We established diabetic and non-diabetic MI mouse models with TFPI2 overexpression (Fig. 4d, e). We found that 3 weeks post MI, MMP2 and MMP9 expression in the infarcted heart was substantially higher in the diabetic group than that in the non-diabetic group (Fig. 4f). Collagen I and III levels were increased in diabetic MI mice compared with those in non-diabetic mice (Fig. 4g). Additionally, the expression levels of MMP2 and 9 and collagen I and III in the diabetic MI mice were substantially reduced with TFPI2 overexpression, which was verified using western blotting (Supplementary Fig. 2 of Additional File 1).

### Overexpression of TFPI2 promotes M1-to-M2 macrophage polarization in diabetic MI mice

**Fig. 5:**
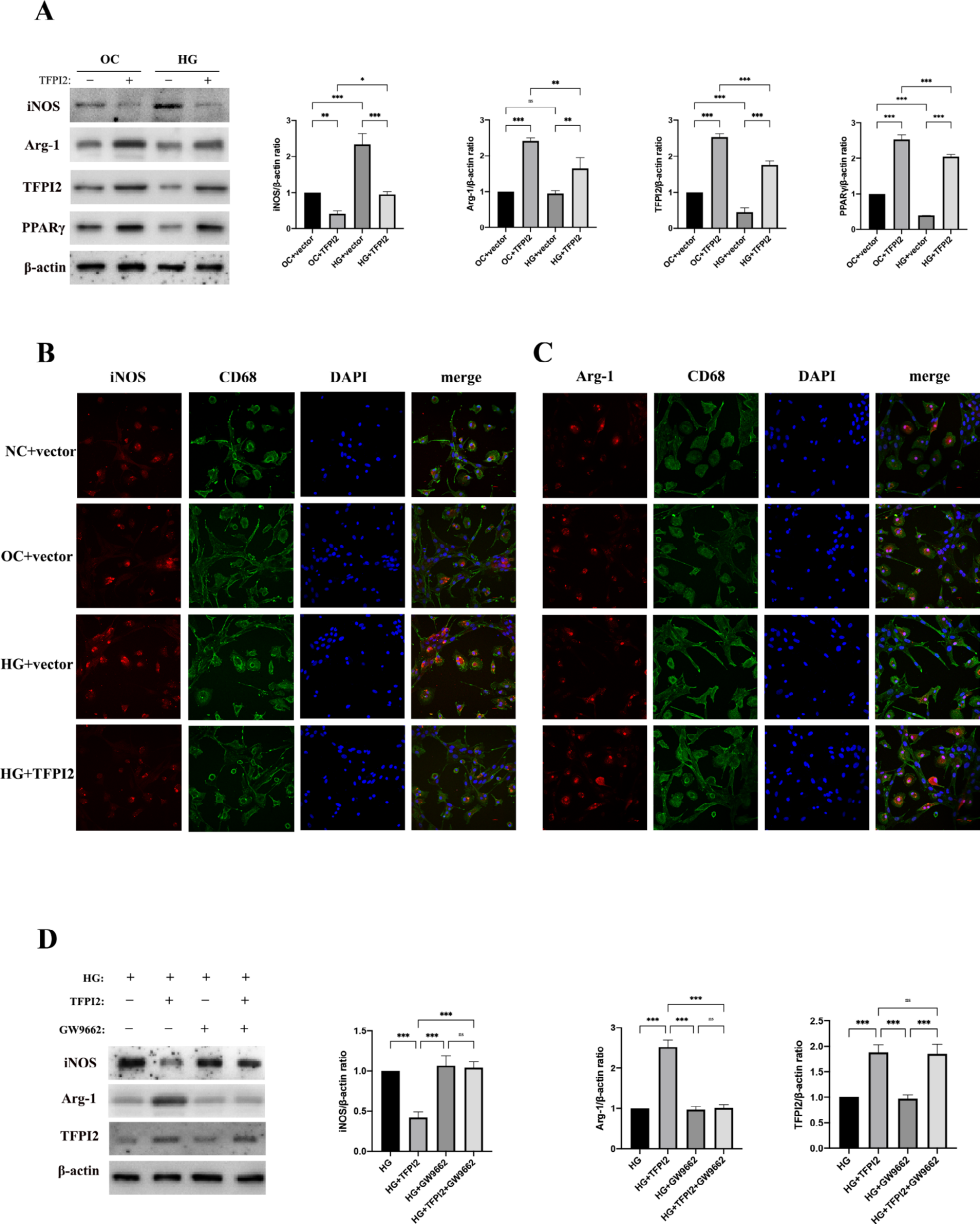
Effects of TFPI2 overexpression on BMDM polarization under high-glucose stimulation (**a**) Western blot analysis of TFPI2, iNOS, Arg-1, and PPARγ in BMDMs (n = 3). (**b**) Co-immunofluorescence staining of the macrophage marker (CD68, green) and M1 marker (iNOS, red) in BMDMs (n = 3, respectively). (**c**) Co-immunofluorescence staining of the macrophage marker (CD68, green) and M2 marker (Arg-1, red) in BMDMs (n = 3). (**d**) Western blot analysis of TFPI2, iNOS, and Arg-1 expression in BMDMs with PPARγ inhibition (n = 3, respectively). NC, normal control; OC, osmotic control; HG, high glucose. TFPI2, transfection with TFPI2 cDNA; vector, transfection with the empty vector. GW9662, PPARγ inhibitor; Data represent mean ± standard deviation (SD). Data were analyzed using one-way ANOVA and Tukey’s post hoc test. ^*^P < 0.05, ^**^P < 0.01, ^***^P < 0.001

To explore the effect of HG stimulation on TFPI2 expression and macrophage polarization in vitro, we isolated BMDMs and transfected them with TFPI2 cDNA before HG treatment. The iNOS expression increased markedly, whereas TFPI2 and PPARγ expression decreased compared with that in the control group. TFPI2 overexpression almost entirely reversed the M1-polarizing effect of HG stimulation and increased Arg-1 and PPARγ expression (Fig. 5a). Immunofluorescence staining of BMDMs also confirmed that TFPI2 overexpression inhibited HG-induced M1 polarization (decreased iNOS expression) (Fig. 5b, Supplementary Fig. 3a of Additional File 1) and promoted the shift to the M2 phenotype (increased Arg-1 expression) (Fig. 5c, Supplementary Fig. 3b of Additional File 1). Next, we explored the involvement of PPARγ in TFPI2-mediated macrophage polarization. Western blot revealed that PPARγ inhibition (GW9662) attenuated the M1-to-M2-polarizing effects of TFPI2 (Fig. 5d).

**Fig. 6:**
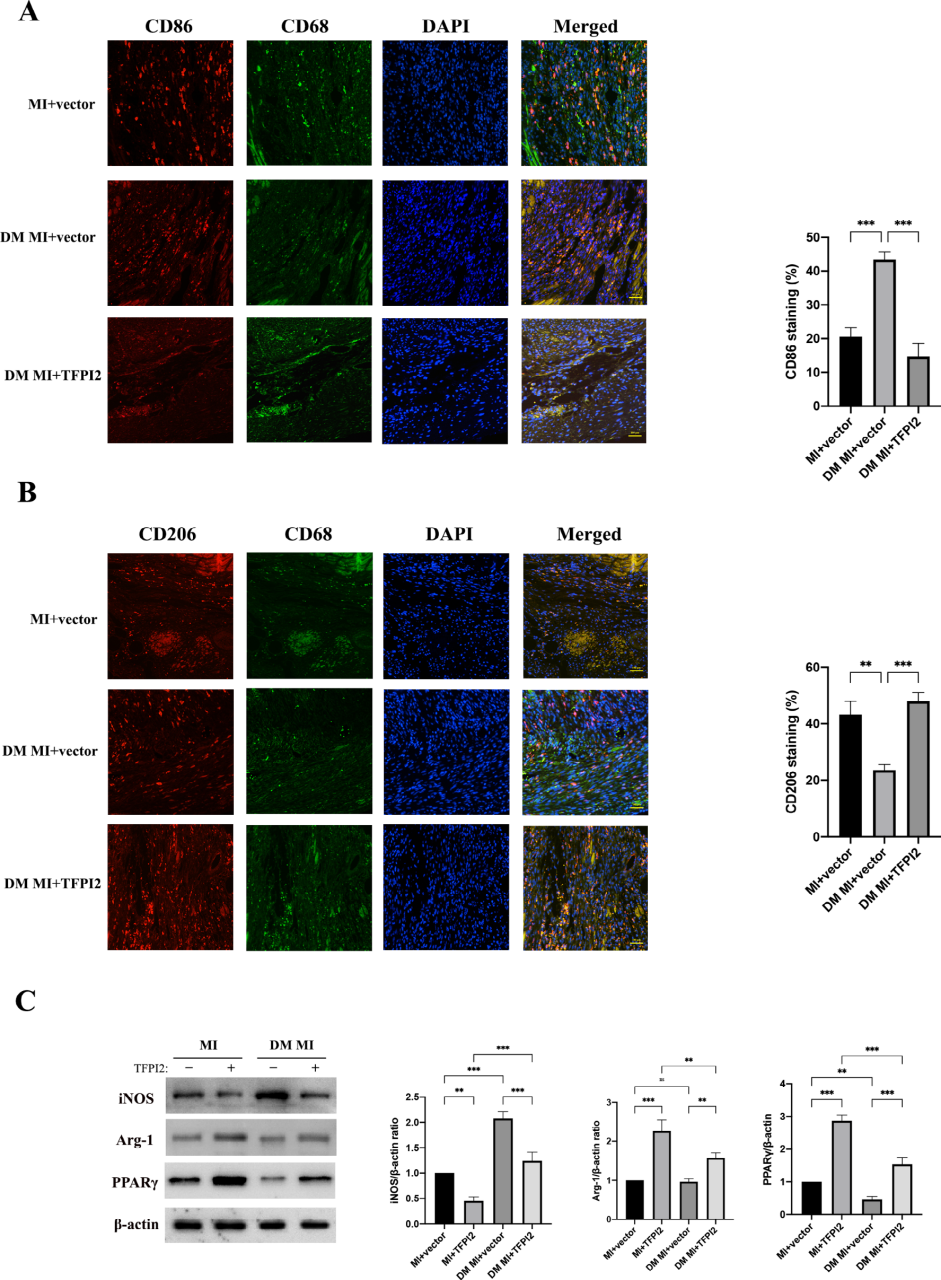
Effects of TFPI2 overexpression on macrophage polarization in diabetic MI mice (**a**) Co-immunofluorescence staining of the macrophage marker (CD68, green) and M1 marker (CD86, red) in the infarcted myocardium samples (n = 3, respectively). (**b**) Co-immunofluorescence staining of the macrophage marker (CD68, green) and M2 marker (CD206, red) in the infarcted myocardium samples (n = 3). (**c**) Western blot analysis of iNOS, Arg-1, and PPARγ at 3 weeks after infarction (n = 3). MI, myocardial infarction; DM, diabetes mellitus; TFPI2, transfection with TFPI2 cDNA; vector, transfection with the empty vector. Data represent mean ± standard deviation (SD). Data were analyzed using one-way ANOVA and Tukey’s post hoc test. ^*^P < 0.05, ^**^P < 0.01, ^***^P < 0.001

The number of CD86^+^ macrophages was substantially higher in the diabetic mice than that in the non-diabetic mice 3 weeks after infarction, whereas the number was substantially lower in the DM MI + TFPI2 group (Fig. 6a). Conversely, only a small number of CD206^+^ macrophages was observed in the infarcted myocardium of diabetic mice, whereas the number was substantially higher in TFPI2-overexpressing mice (Fig. 6b). Western blot analysis also confirmed that TFPI2 overexpression inhibited hyperglycaemia-induced M1 macrophage polarization and promoted transition to reparative M2 macrophages (Fig. 6c).

### Overexpression of TFPI2 promotes cardiac function recovery in diabetic mice

**Fig. 7:**
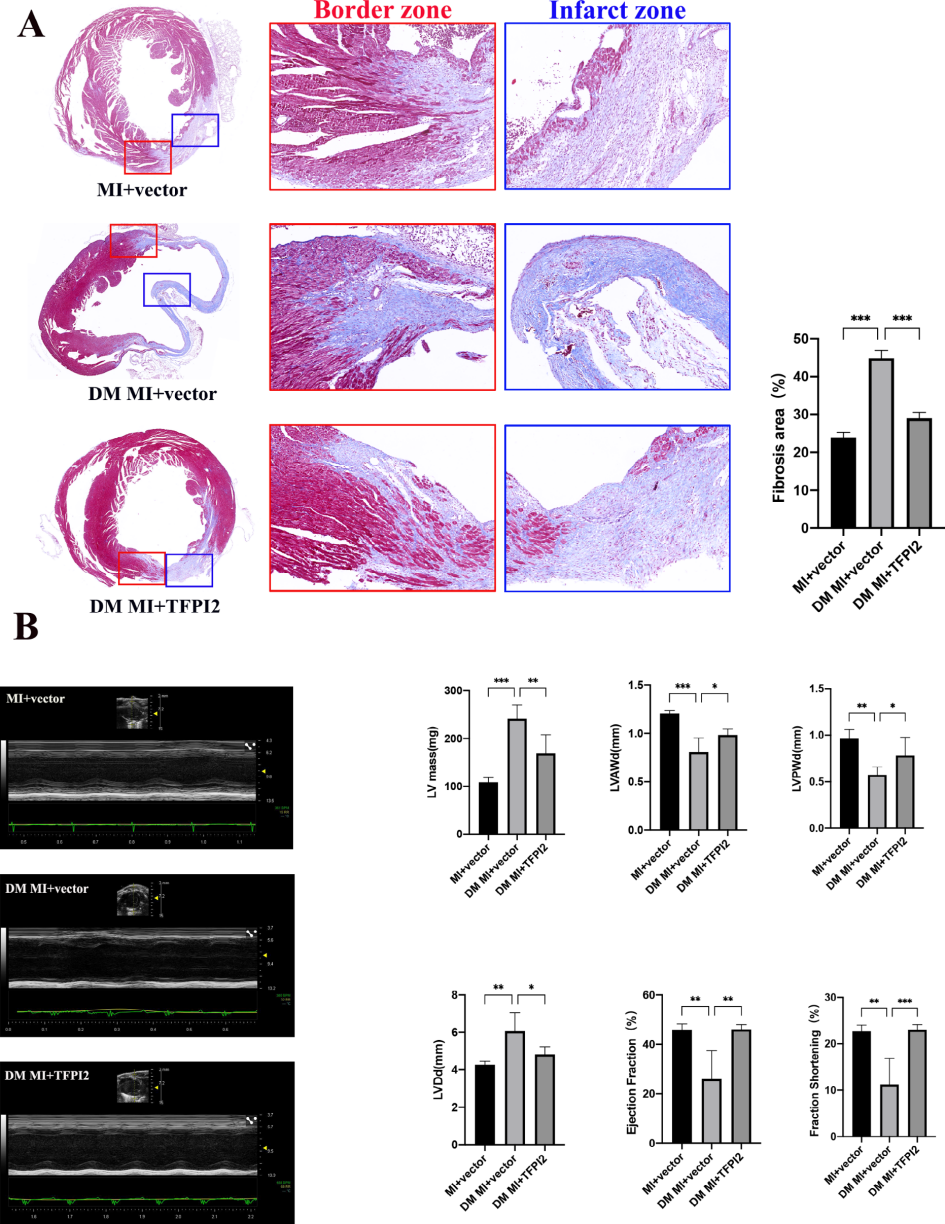
Effects of TFPI2 overexpression on post-MI remodeling and cardiac function recovery in diabetic mice (**a**) Representative Masson’s trichome staining images of the infarct and border zones of post-MI myocardium samples and their quantitative analysis (n = 3). (**b**) Quantitative analysis of cardiac function recovery using echocardiography (n = 4). MI, myocardial infarction; DM, diabetes mellitus; TFPI2, transfection with TFPI2 cDNA; vector, transfection with the empty vector. Data represent mean ± SD. Data were analyzed using one-way ANOVA and Tukey’s post hoc test. ^*^P < 0.05, ^**^P < 0.01, ^***^P < 0.001

The area of fibrosis in the MI tissue was substantially larger in the diabetic mice than in the non-diabetic mice but was smaller in the DM MI + TFPI2 group, as indicated by Masson’s trichome staining (Fig. 7a). The diabetic mice exhibited reductions in LVAWd and LVPWd, an enlargement of LVDd and LV mass, and markedly lower EF and FS, 3 weeks after infarction than the non-diabetic, control mice. TFPI2 overexpression resulted in increased LVAWd and LVPWd, reductions in LVDd and LV mass, and a significant improvement in EF and FS; therefore, a substantial functional recovery was observed in the DM MI + TFPI group (Fig. 7b).

## Discussion

The present study provided the following novel insights into the relationship among hyperglycaemia, TFPI2, and macrophage polarization during the post-MI healing process: (1) TFPI2 downregulation was at least partially responsible for the poor post-MI cardiac remodelling and reduced cardiac function in diabetic mice; (2) TFPI2 promoted M1-to-M2 polarization via PPARγ activation, but hyperglycaemia skewed macrophage phenotype polarization toward M1 by downregulating TFPI2 expression, leading to prolonged post-MI inflammation; and (3) TFPI2 overexpression attenuated HG-induced CF migration and collagen synthesis, thereby improving post-MI remodelling and cardiac function recovery.

In the present study, we confirmed that TFPI2 was substantially downregulated in the infarcted myocardium under diabetic conditions and revealed the protective effect of TFPI2 on post-MI remodelling. As a broad-spectrum serine protease inhibitor [25], TFPI2 is reportedly secreted in the ECM, suppressing ECM degradation by either directly inhibiting MMP activity or indirectly inhibiting plasmin- and trypsin-mediated MMP activation [19, 26–28]. TFPI2 is also widely distributed in the cytoplasm and nucleus, and nuclear-localized TFPI2 can interact with the transcription factor AP-2α and thereby negatively regulate the transcription and expression of MMP2 [23]. In cardiovascular diseases, previous studies have indicated that TFPI2 can inhibit hyperglycaemia-induced vascular smooth muscle cell (VSMC) migration and neointimal hyperplasia by inhibiting the expression and activity of MMPs [17, 29]. In the present study, we found that TFPI2 overexpression inhibited MMP2 and MMP9 expression in the infarcted myocardium, suppressing ECM degradation, inflammatory infiltration, and myofibroblast migration and activation, thereby attenuating excessive collagen deposition and adverse fibrosis remodelling, which is in accordance with previous studies [30, 31]. Understanding this pathway provides important molecular insights into the role of TFPI2 in poor post-MI remodelling under diabetic conditions.

TFPI2 could promote macrophage polarization toward the M2 phenotype and attenuated the inflammatory response by activating PPARγ. Consistently, conditional knockout of TFPI2 in vascular endothelial cells was reportedly to inhibit the activation of PPARα and PPARγ, thereby contributing to atherosclerotic plaque development [20].Our previous study revealed that PPARγ activation could promote M1-to-M2 macrophage phenotype transition under diabetic conditions [32]. The essential role of PPAR-γ in phenotypic changes can be partially attributed to the induction of fatty acid oxidation in macrophages [33–35]. However, the molecular mechanism underlying the TFPI2/PPARγ pathway remains to be explored. The transcription factor, AP-2α, can inhibit PPARγ expression during adipogenesis, probably via H3K9me3 [36]. TFPI2 was reported to translocate into the nucleus and interact with AP-2α, thereby attenuating the binding of AP-2α to its target gene [23]. Thus, we propose that TFPI2 may upregulate PPARγ expression by interacting with AP-2α.

As an imprinted gene cluster, the promoter of TFPI2 contains a 220-bp long CpG island region that spans exon 1 and three transcription initiation sites [37]. Aberrant DNA methylation of the CpG island region, i.e., the binding of a methyl group to the cytosine 5-carbon covalent bond of CpG dinucleotides, is associated with transcriptional silencing of *TFPI2*. Previous studies have reported that TFPI2 is frequently silenced by aberrant hypermethylation of its promoter in several tumour tissues [38–41]. In our previous study, we discovered that TFPI2 was downregulated in hyperglycaemia-stimulated VSMCs, in which PARP1 activation plays a key role in promoting DNA methylation of the *TFPI2* promoter and TFPI2 downregulation [17]. Li et al. [42] showed that the level of DNA methylation was much higher during myocardial ischemia/reperfusion injury under diabetic conditions than under nondiabetic conditions. Thus, we propose that TFPI2 downregulation in the infarcted heart under diabetic conditions is related to DNA hypermethylation of the *TFPI2* promoter.

There are some limitations to this study: (1) Both macrophage polarization and fibroblast activation and migration function as a double-edged sword during post-MI healing. Herein, we solely focused on studying the effects of TFPI2 overexpression or knockdown on cardiac remodelling and function recovery 3 weeks after infarction, whereas their effects on long-term prognosis remain to be determined; (2) whether TFPI2 downregulation in the infarcted heart is related to DNA hypermethylation of the *TFPI2* promoter under diabetic conditions and the underlying molecular mechanisms remain to be determined; and (3) the molecular mechanism of TFPI2 regulating PPARγ expression remains to be explored.

## Conclusions

Downregulation of TFPI2 under hyperglycaemic conditions contributes to adverse cardiac remodelling and poor functional recovery after MI. TFPI2 upregulation or activation may be a promising therapeutic strategy for early resolution of post-MI inflammation and excessive fibrotic remodelling under diabetic conditions.

## Methods

### Type 1 diabetes mouse model

We used 6-week-old male C57BL/6 mice (20–25 g) purchased from SPF (Beijing) Biotechnology Co., Ltd. [License No.: SCXK (Beijing) 2019–0010] in this study. The mice were housed in a temperature-controlled environment (21 ± 2 °C) with a 12-h light/dark cycle (lights on at 06:00) and *ad libitum* access to food and water. To establish the type I diabetes mouse model, the mice (n = 12) were intraperitoneally administered streptozotocin (STZ) (50 mg/kg) in citrate buffer (0.05 mol/L; pH 4.5) for 5 d; control mice (n = 30) received an equivalent amount of citrate buffer solvent. Two weeks after the initial administration of STZ, the mice with ≥ 16.7 mM (300 mg/dL) blood glucose level were considered diabetic and included in the diabetic cohort [43].

### MI mouse model

The mice were anesthetized using isoflurane (2%; O_2_ 2 L/min), and the surgery was performed after confirming that the pedal reflexes of the mice were absent. The mice underwent left thoracotomy at the fourth intercostal space; their hearts were smoothly and gently extracted, and the left anterior descending branch of the coronary artery was distally ligated to its main bifurcation using a 7 − 0 ophthalmic suture. The success of the coronary occlusion was confirmed based on the pallor and regional-wall motion abnormality of the left ventricle or ST-segment elevation ≥ 0.25 mV on an ECG monitor [44]. Sham mice underwent the same time-matched surgical procedure without a ligation step. For cardiac-specific expression or silencing of TFPI2 in the MI mice, adenovirus (2 × 10^9^ plaque-forming units (PFU/L, 30 µL) was directly injected into the myocardium at three positions along the margin of the ischemic area when inducing MI; another 2 × 10^9^ PFU/L of adenovirus was injected into the tail vein after 1 week [45, 46]. Each mouse was injected with adenovirus carrying TFPI2 cDNA or the empty vector, sh-TFPI2, or sh-NC.

Consequently, there are seven groups of mice in this study (n = 6 for each group), including Sham group, MI mice with sh-TFPI2 knockdown (MI + sh-TFPI2), MI mice transfected with sh-NC (MI + sh-NC), MI mice with TFPI2 overexpression (MI + TFPI2), MI mice transfected with empty vector (MI + vector), diabetic MI mice with TFPI2 overexpression (DM MI + TFPI2), and diabetic MI mice transfected with cDNA vector (DM MI + vector). The mice were euthanized at 3 weeks post MI according to their groups. The study protocol was approved by the Ethics Committee of Qingdao University School of Medicine (QYFY WZLL 27,656, Qingdao, China).

### Echocardiography

In the 3rd week after MI surgery, transthoracic echocardiography was performed using the Vevo2100 imaging system (Visual Sonics, Toronto, Canada). Moreover, 2D echocardiography and M-mode echocardiography were used to measure EF, LV, FS, LVDd, LVAWd, and LVPWd. All measurements were performed by the same observer, and the values were averaged over five consecutive cardiac cycles [47].

### Cell culture and treatment

BMDMs were isolated from C57BL/6 mice and cultured in Dulbecco’s modified Eagle medium (DMEM) containing 20 ng/mL mouse macrophage-colony-stimulating factor for 5–7 d to induce M0 macrophages. CFs were isolated by enzymatic digestion and cultured in full DMEM as previously described [16]. The CFs isolated from male neonatal C57BL/6J mice (1–3 d) were cultured under normal glucose conditions with 10% foetal bovine serum till the second generation before treatment.

Next, the cells were transfected with TFPI2 cDNA or the empty vector, sh-TFPI2, or sh-NC, following which the cells were incubated in a medium with 5 mM D-glucose (normal control, NC), 5 mM D-glucose + 27.5 mM mannose (osmotic control, OC), 33 mM D-glucose (HG), or 33 mM D-glucose + 3 µM PPARγ antagonist (GW9662, Selleck Chemicals, United States) for 48 h [48].

### Histopathology and immunofluorescence analyses

The mouse hearts were harvested and fixed in 4% formalin for at least 24 h, followed by paraffin embedding and sectioning (5 μm). Inflammatory cell infiltration was assessed using haematoxylin-eosin staining (H&E), and the extent of post-infarct fibrosis was assessed using Masson’s trichrome staining. For immunohistochemistry, the slides were incubated overnight at room temperature with primary antibodies against collagen I (1:50, ab138492, Abcam), collagen III (1:50, ab184993, Abcam), MMP2 (1:50, ab92536, Abcam), and MMP9 (1:50, ab283575, Abcam). For immunofluorescence analysis, the sections were simultaneously labelled using unconjugated primary antibodies against CD68 (1:200, 14-0681-82, eBioscience) and the M1 markers CD86 (1:200, ab242020, Abcam)/iNOS (1:500, ab129372; Abcam), or M2 markers CD206 (1:200, ab64693, Abcam)/Arg-1(1:500, ab91279; Abcam) and incubated overnight, followed by incubation with a fluorophore-conjugated secondary antibody for 30 min. The stained sections were mounted using DAPI-containing Vector Shield mounting medium (Vector). All pathological sections were scanned and photographed using the Qingdao University Hospital Digital Cross Section Scanning System. Data were collated for analysis using the ImageJ software.

### Western blotting

The cells or extracted heart tissue samples were homogenized using RIPA lysis buffer (Elabscience) and centrifuged at 15,000 × *g* for 10 min at 4 °C. The supernatant was collected, and the protein concentration was determined using a BCA assay kit (Thermo Fisher Scientific). The proteins were separated using 10% sodium dodecyl sulphate polyacrylamide gel electrophoresis and transferred onto nitrocellulose membranes (Merck Millipore, USA). The membranes were blocked with 5% skim milk for 2 h and incubated overnight at 4 °C with the primary antibodies against the following proteins: TFPI2 (1:1000, ab186747; Abcam), iNOS (1:1000, ab129372; Abcam), Arg-1 (1:1000, ab91279; Abcam), PPAR-γ (1:1000, Santa Cruz), collagen I (1:50, ab138492, Abcam), collagen III (1:50, ab184993, Abcam), MMP2 (1:50, ab92536, Abcam), MMP9 (1:50, ab283575, Abcam), and β-actin (1:4000, 8H10D10, CST). Horseradish peroxidase (HRP)-labelled goat anti-rabbit IgG (1:10,000, Absin, Shanghai, China) was used as the secondary antibody and incubated for 1 h at room temperature. Target bands were visualized using chemiluminescent ECL (Merck Millipore, USA) and detected using an Amersham Imager 600 (GE Healthcare, Little Chalfont, UK). The images were analysed using the ImageJ data acquisition software.

### Cell migration analysis

We investigated cell migration using wound healing and Transwell assays. For the wound healing assay, CFs were seeded in six-well plates. Wounds were made through the cell monolayer using 1000-mL plastic tips after the cells were incubated for 12 h, and images were captured using a Nikon Ti-S inverted phase-contrast microscope (Nikon, Tokyo, Japan) at 24 h to calculate the healing rates.

For the Transwell assay, the cells (1 × 10^5^ cells) were trypsinized and seeded into the upper chambers (8-mm pores, 24-well, Corning Life Sciences, Corning, NY, USA) in FBS-free DMEM (200 mL). The lower chambers contained DMEM supplemented with HG or the OC, and incubation was performed for 24 h. Next, the cells that migrated into the lower chambers were fixed using 4% paraformaldehyde after removal of the medium and stained with crystal violet for 0.5 h. Images of five stochastic fields per membrane were obtained using a Nikon Ti-S inverted phase-contrast microscope.

### Statistical analysis

All experiments were repeated at least three times, and data are presented as mean ± SD (standard deviation). Statistical analysis was performed using ANOVA followed by Tukey’s post hoc test (GraphPad Prism 9, USA). *P* < 0.05 was considered significant.

### Electronic supplementary material

Below is the link to the electronic supplementary material.

Supplementary Material 1

Supplementary Material 2

Supplementary Material 3

Supplementary Material 4

Supplementary Material 5

## Data Availability

All data generated or analysed during this study are included in this published article and its additional information files.

## Abbreviations

MI: Myocardial infarction
ECM: Extracellular matrix
DM: Diabetes mellitus
MMPs: Matrix metalloproteinases
CFS: Cardiac fibroblasts
TFPI2: Tissue factor pathway inhibitor-2
LPS: Lipopolysaccharide
PPARγ: Peroxisome proliferator-activated receptor gamma
LVAWd: Left ventricular end-diastolic anterior wall thickness
LVPWd: Left ventricular end-diastolic posterior wall thickness
LV: Left ventricular
LVDd: Left ventricular end-diastolic internal dimension
EF: Ejection fraction
FS: Fraction shortening
HG: High-glucose
VSMC: Vascular smooth muscle cell
H&E: Haematoxylin-eosin
STZ: Streptozotocin
AGEs: Advanced glycation end products
iNOS: Inducible nitric oxide synthase
Arg-1: Arginase-1
