# Supplementary figures and images for: The TFPI2–PPARγ axis induces M2 polarization and inhibits fibroblast activation to promote recovery from post-myocardial infarction in diabetic mice
Source: J Inflamm (Lond). 2023 Nov 1;20:35. doi: 10.1186/s12950-023-00357-8 (PMC10621166; doi:10.1186/s12950-023-00357-8)

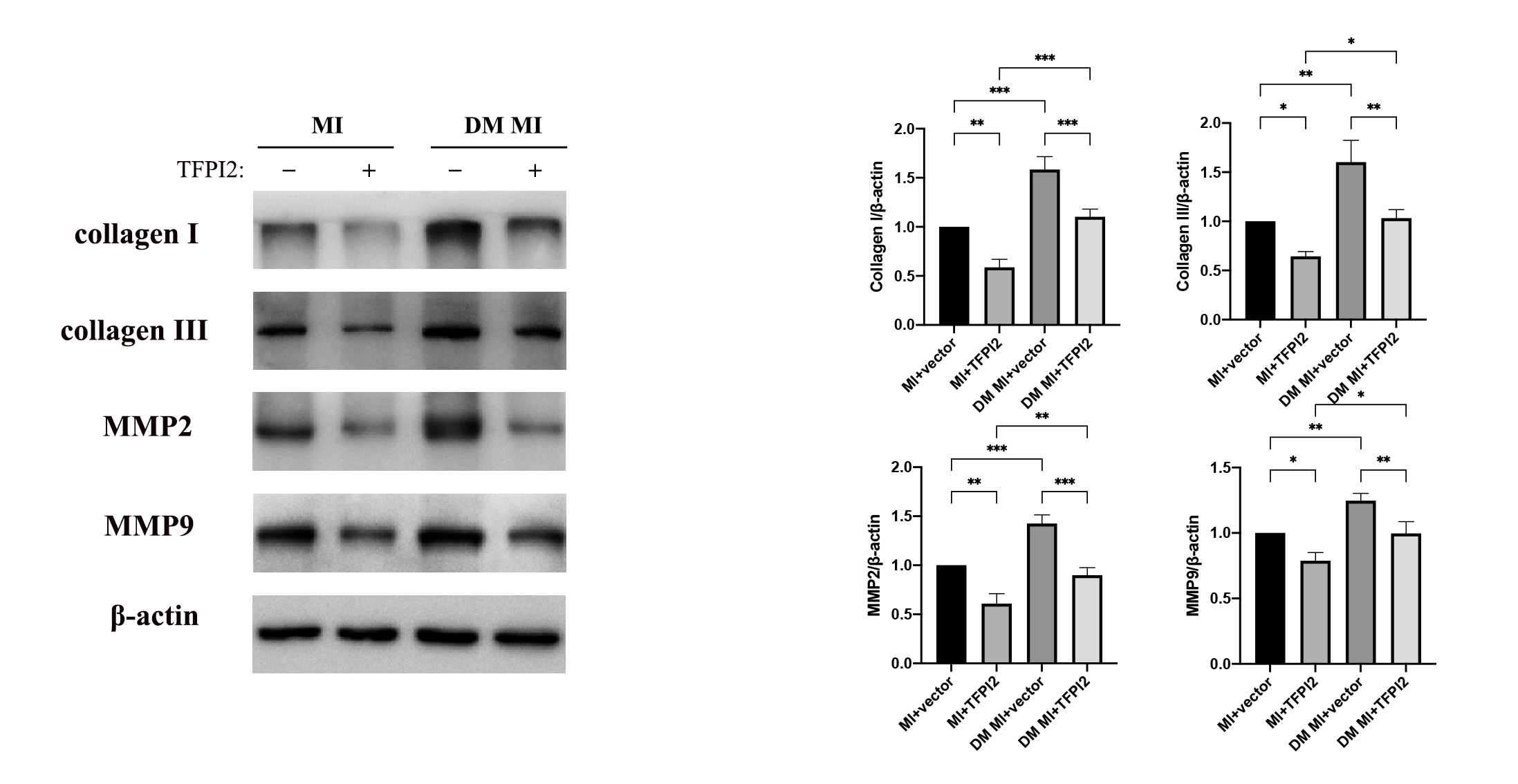

Supplement: Supplementary file 1 — Supplementary Material 1 [file 12950_2023_357_MOESM1_ESM.png]

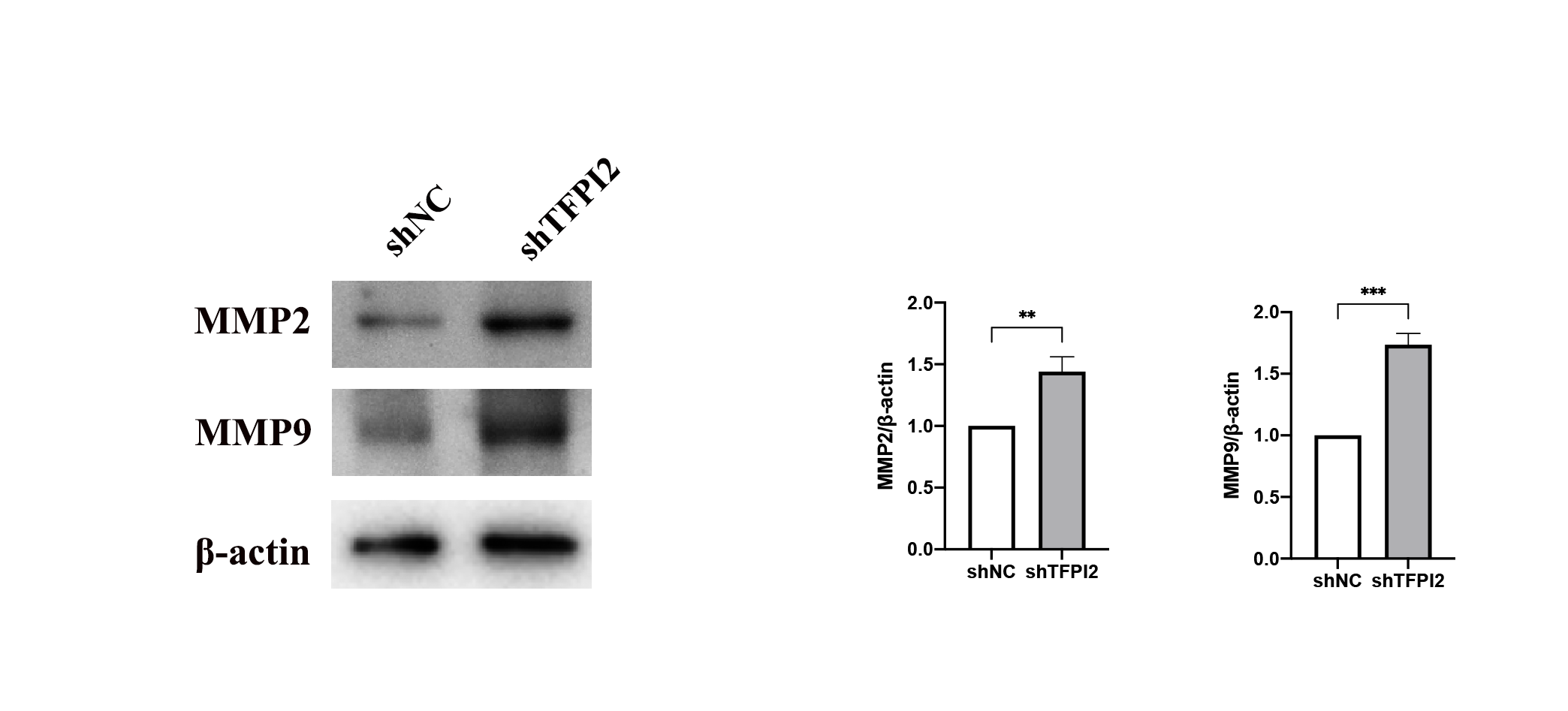

Supplement: Supplementary file 3 — Supplementary Material 3 [file 12950_2023_357_MOESM3_ESM.png]

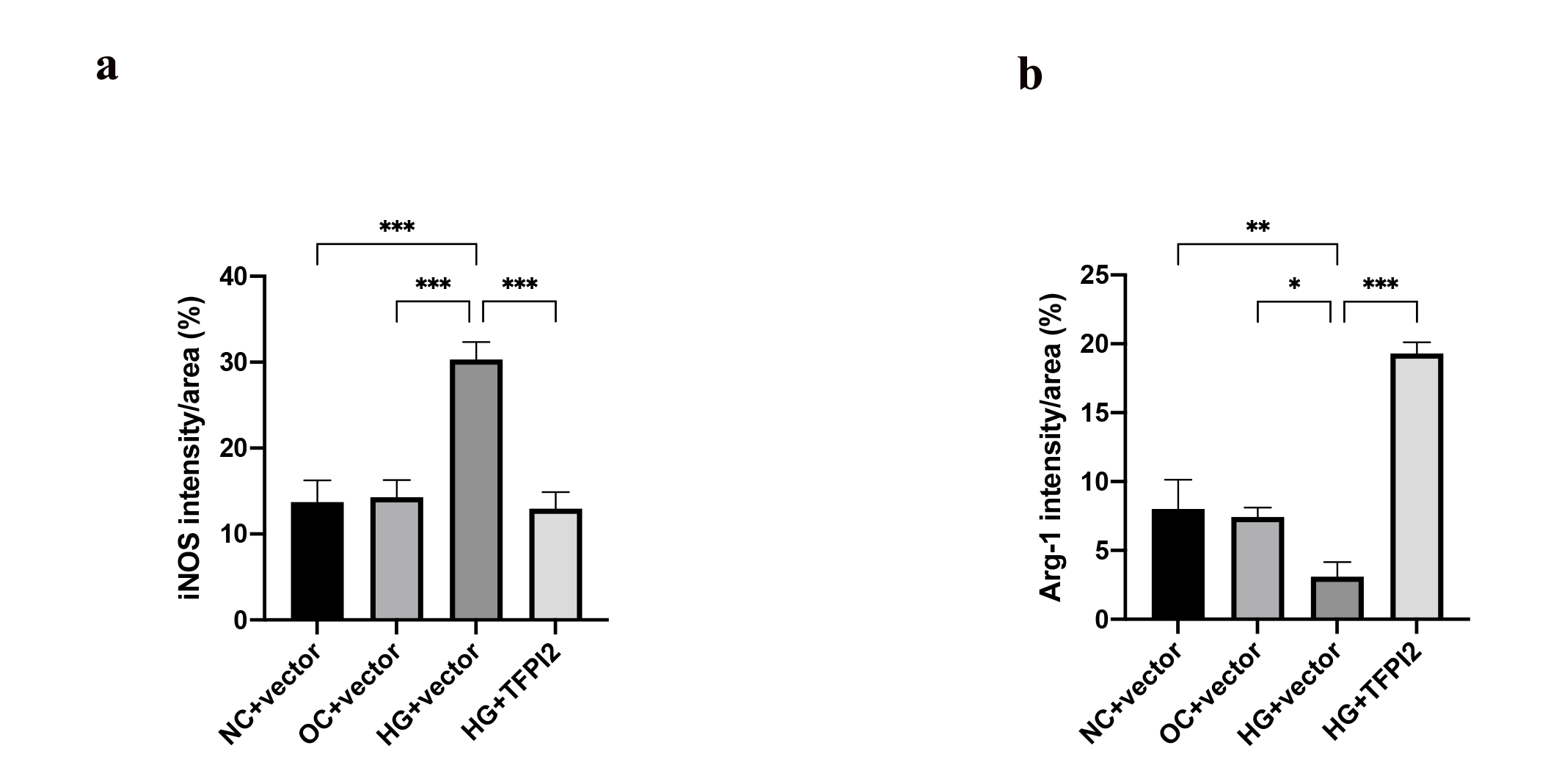

Supplement: Supplementary file 4 — Supplementary Material 4 [file 12950_2023_357_MOESM4_ESM.png]
