# Supplementary material for: The TFPI2–PPARγ axis induces M2 polarization and inhibits fibroblast activation to promote recovery from post-myocardial infarction in diabetic mice
Source: J Inflamm (Lond). 2023 Nov 1;20:35. doi: 10.1186/s12950-023-00357-8 (PMC10621166; doi:10.1186/s12950-023-00357-8)
Supplement: Supplementary file 2 — Supplementary Material 2 [file 12950_2023_357_MOESM2_ESM.docx]

**Additional file 1**

**Supplementary Figure 1**

Western blot analysis of MMP2 and MMP9 in bone marrow-derived macrophages (BMDMs) transfected with sh-TFPI2 (n = 3, respectively). sh-NC, sh-NC transfection; sh-TFPI2, sh-TFPI2 transfection. Data represent mean ± standard deviation (SD). Data were analyzed using one-way ANOVA and Tukey’s post hoc test. ^*^*P* < 0.05, ^**^*P* < 0.01, ^***^*P* < 0.001.

**Supplementary Figure 2**

Western blot analysis of MMP2, MMP9, collagen I, and collagen III in the infarcted heart (n = 3). Sham, sham operation; MI, myocardial infarction; DM, Diabetes mellitus; TFPI2, transfection with TFPI2 cDNA; vector, transfection with the empty vector; Data represent mean ± SD. Data were analyzed using one-way ANOVA and Tukey’s post hoc test. ^*^*P* < 0.05, ^**^*P* < 0.01, ^***^*P* < 0.001.

**Supplementary Figure 3**

(a) Quantitative analysis of macrophage markers and M1 markers (iNOS) in bone marrow-derived macrophages (BMDMs) by co-immunofluorescence staining (n = 3). (b) Quantitative analysis of macrophage markers and M2 markers (Arg-1) in BMDMs by co-immunofluorescence staining (n = 3). NC, normal control; OC, osmotic control; HG, high glucose. Data represent mean ± SD. Data were analyzed using one-way ANOVA and Tukey’s post hoc test. ^*^*P* < 0.05, ^**^*P* < 0.01, ^***^*P* < 0.001.
