# Supplementary material for: The TFPI2–PPARγ axis induces M2 polarization and inhibits fibroblast activation to promote recovery from post-myocardial infarction in diabetic mice
Source: J Inflamm (Lond). 2023 Nov 1;20:35. doi: 10.1186/s12950-023-00357-8 (PMC10621166; doi:10.1186/s12950-023-00357-8)

Fig1

TFPI2 β-actin

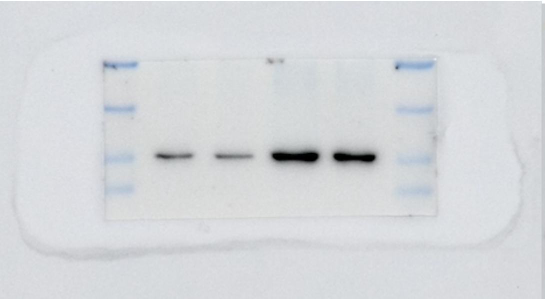
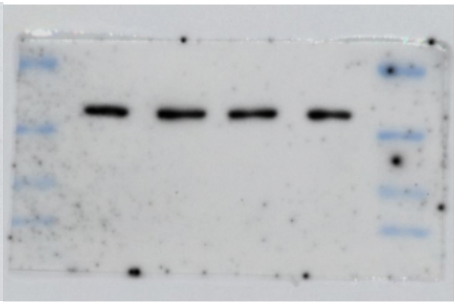

TFPI2 β-actin

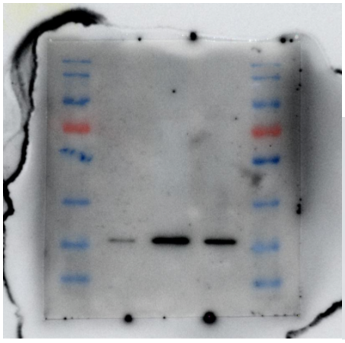
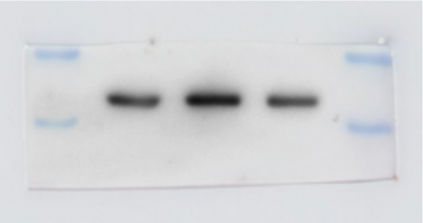

Fig2

MMP2 MMP9 β-actin

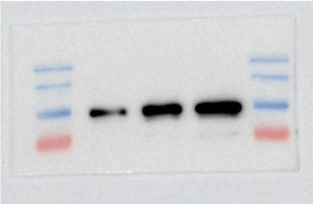
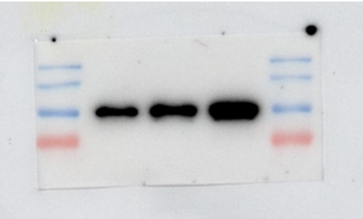
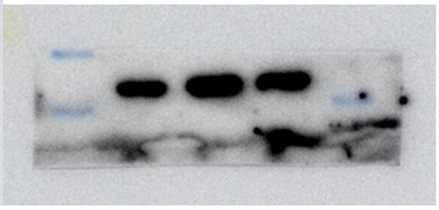

MMP2 MMP9 β-actin

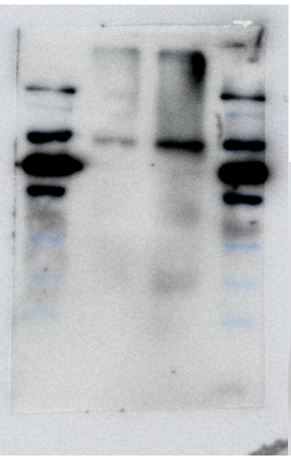
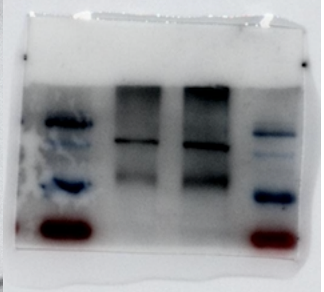
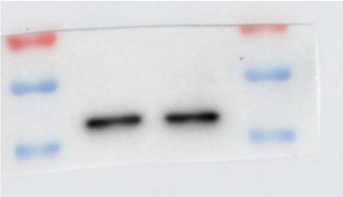

Fig3

iNOS Arg1 PPARγ β-actin

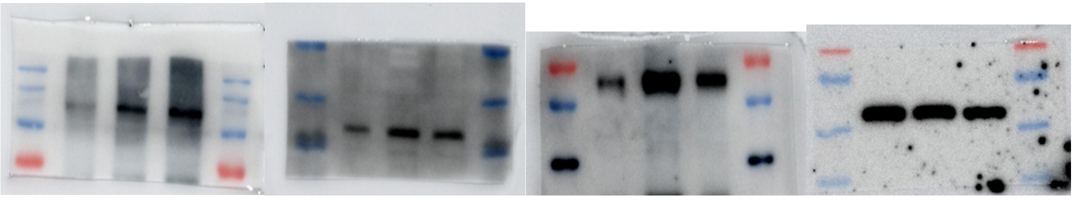

Fig4

Col 1 Col 3 MMP2 MMP9 β-actin

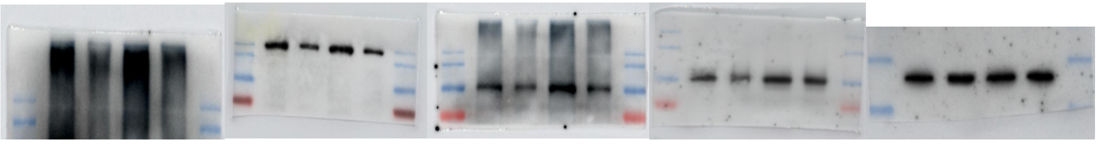

TFPI2 β-actin

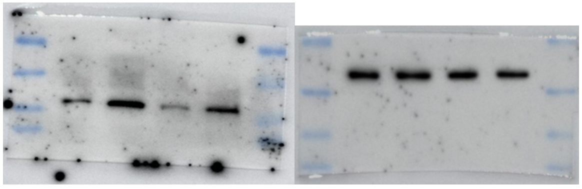

Fig5

iNOS Arg1 TFPI2 PPARγ β-actin

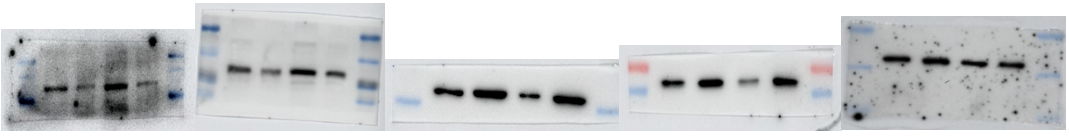

iNOS Arg1 TFPI2 β-actin

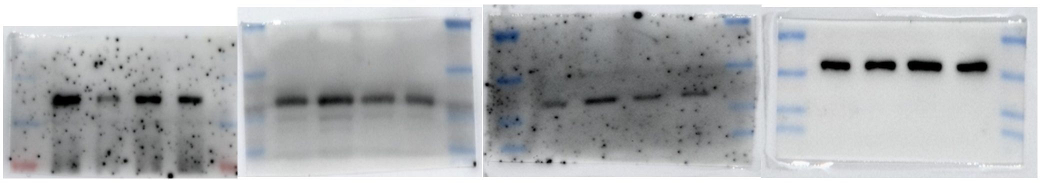

Fig6

iNOS Arg1 PPARr β-actin

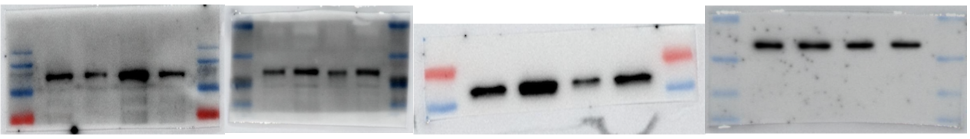

Sup 1

MMP2 MMP9 β-actin

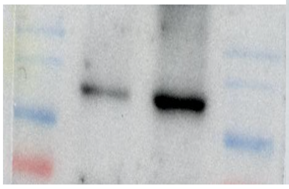

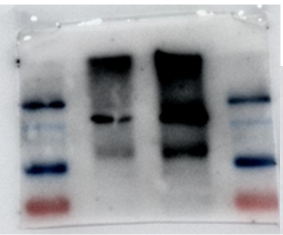

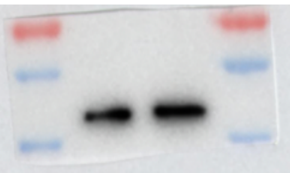

Sup 2

Col 1 Col 3

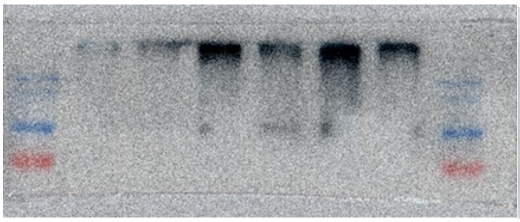

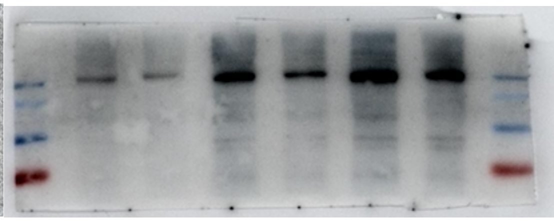

MMP2 MMP9

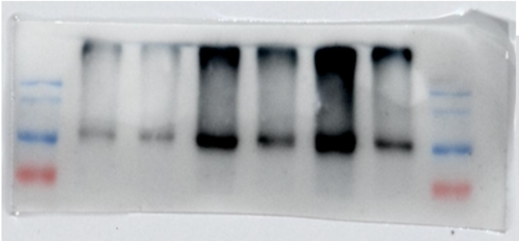

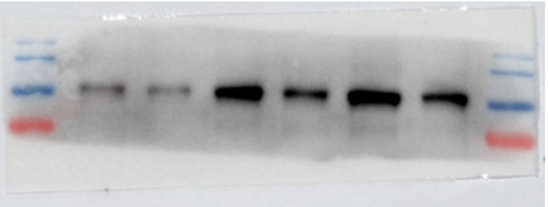

β-actin

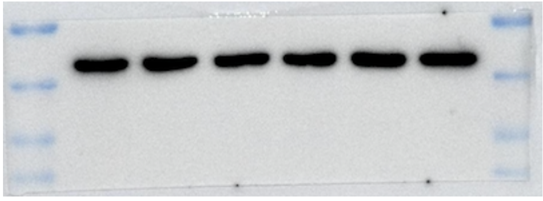

Supplement: Supplementary file 5 — Supplementary Material 5 [file 12950_2023_357_MOESM5_ESM.pdf]
